# Preparing children's nurses for working with children and adolescents who self‐harm: Evaluating the ‘*our care through our eyes*’ e‐learning training package

**DOI:** 10.1111/inm.13041

**Published:** 2022-07-16

**Authors:** Jasmine Singh‐Weldon, Vicki Tsianakas, Trevor Murrells, Annmarie Grealish

**Affiliations:** ^1^ Florence Nightingale Faculty of Nursing, Midwifery & Palliative Care King's College London London UK; ^2^ Department of Nursing University of Limerick Limerick Ireland

**Keywords:** adolescent, child, e‐learning, nurse education, self‐harm

## Abstract

Rates of self‐harm among children and adolescents have risen significantly over the past decade and clinical guidelines place children's nurses at the heart of their care. This article reports on the evaluation of ‘Our Care Through Our Eyes’, an online self‐harm learning programme for children's nurses. A self‐selected, convenience sample of registered children's nurses (*n* = 42) completed scales pre‐ and postlearning programme that captured their attitudes, beliefs, empathy, anxiety, and confidence. Mean change scores were assessed, and qualitative comments captured postintervention were thematically summarized. There were small improvements in participants' attitudes, empathy and confidence were reported. Anxiety scores increased in a small number of items. Qualitative comments confirmed the value of the online learning programme for improving children's nurses' knowledge and understanding of self‐harm among CYP. Our findings demonstrate that children's nurses agree on the importance of mental health training in self harm, and this could be a catalyst for renewal of both pre‐ and postregistration education including support structures within the National Health Service. This study is the first to explore the feasibility of evaluating ‘Our Care Through Our Eyes’ delivered using e‐leaning and could be used to inform further investigations.

## INTRODUCTION

The National Institute for Health and Care Excellence makes recommendations for the management of children and young people (CYP) who present to an emergency department following an episode of self‐harm (NICE 2004). Initial triage, assessment and management should be undertaken by suitably trained healthcare professionals, and once risks have been established and appropriate interventions decided, all CYP under the age of 16 years should be admitted *‘overnight to a paediatric ward and fully assessed the following day before discharge or further treatment and care is initiated’* (Nice 2004, p28). Between 2010/11 and 2017/18 CYP attendances at accident and emergency (A&E) services following self‐harm increased by 20% (UK Parliament 2018). Although children's nurses are at the forefront of caring for an increasing number of CYP who self‐harm, evidence suggests the profession feels ill‐prepared to provide the necessary mental health care (Bolland *et al*. 2017).

## BACKGROUND

One in six (16%) children between 5 and 16 years of age had a probable mental illnesses in 2020; this represents a 5% rise from one in nine children in 2017 (NHS Digital 2020). The survey was conducted in July 2020 during the Covid‐19 pandemic and those with a probable mental illnesses were significantly more likely to report a worsening of their lives as a consequence of lockdown (NHS Digital 2020). Although 50% of mental health illnesss are established by the age of 14 and 75% by age 24 (Kessler *et al*. 2005), services have been criticized for not intervening until complex symptoms and potentially serious conditions arise. In 2017, Frith (2017) estimated that over one quarter of those referred to Child and Adolescent Mental Health Services (CAMHS) were not accepted for care. An over‐stretched service sector means that opportunities for ‘upstream’ preventative interventions are often missed. This situation is likely to persist as the public sector grapples with the consequences of the Covid‐19 pandemic.

Children and young people with poor mental health may self‐harm as a way of managing or expressing feelings and painful memories (Hawton *et al*. 2012; Rowe *et al*. 2014). Estimates vary from one in fifteen 11–25 year olds (Geulayov *et al*. 2018) to one in five 15 year olds (Public Health England 2017) who report ever having self‐harmed. Adolescent self‐harm has increased over the past decade, particularly among 13–16 year old girls, rising by 68% since 2011 (Morgan *et al*. 2017). In 2017/18 a staggering 21 904 children and young people were hospitalized due to self‐harm injury (UK Parliament 2018), and between 2012/13 and 2018/19 hospital admissions of women aged 10–24 years following self‐harm rose by 36% (Nuffield Trust 2020). These statistics emphasize the importance of non‐CAMHS disciplines having the necessary skills to identify, assess, and manage mental health difficulties in CYP, including children's nurses.

Evidence suggests that children's nurses feel ill‐prepared to provide mental health care for CYP. They report not knowing what to do or say (Richardson 2011), find it difficult to communicate effectively (Bolland *et al*. 2017), and worry about saying the wrong thing and making things worse (Lowe & Campbell 2014). A systematic review of adult nurses' attitudes towards caring for patients who self‐harm reported negative feelings of irritation, frustration and anger (Karman *et al*. 2015). More specifically, the Mental Health Foundation (2015) has highlighted the stigma and discrimination that young people with mental health difficulties can face. Without up‐to‐date knowledge and support, children's nurses may also lack the understanding and empathy necessary to provide holistic care. These strands of the literature highlight the need for training to develop children's nurses' knowledge, skills and confidence to care for CYP who self harm.

Two pilot studies with favourable results have examined the impact of training on children's nurses' knowledge and understanding of mental health, one of which was specifically for self‐harm (Higson *et al*. 2017; Manning *et al*. 2017). Manning *et al*. (2017) designed and implemented the self‐harm online training programme ‘Our Care Through Our Eyes’ that employed reusable learning objects (RLOs). That is digital learning resources with a single learning objective that engage the user in four learning components. Following implementation of the RLO's, improvements in knowledge, attitudes, confidence and clinical behaviours were identified, and the children's nurses also reported feelings of empowerment and improved communication skills (Manning *et al*. 2017).

Increasing rates of adolescent self‐harm (Morgan *et al*. 2017) and associated emergency department attendances (Nuffield Trust 2020), combined with the requirement that those under the age of 16 years should be admitted to a paediatric ward (NICE 2004), underline the central role of the children's nurse. The workforce needs training to facilitate their support of CYP who are admitted following an episode of self‐harm. Training initiatives for children's nurses have shown positive results and there is an opportunity to build on the RLO pilot work of Manning *et al*. (2017) by applying it in a different setting and capturing a different set of impact indicators. This article reports on a study that aimed to evaluate how the ‘Our Care Through Our Eyes’ RLO training programme influenced children's nurses' attitudes, beliefs, empathy, confidence, and anxiety in relation to the care of CYP who self‐harm.

## METHODS

### Design

A single group, pre–posttest design was used to assess the efficacy of the RLO intervention. The study is reported in accordance with the STrengthening the Reporting of OBservational studies in Epidemiology (STROBE) statement for cohort studies (von Elm *et al*. 2007).

### Setting and intervention

The study was conducted in a large inner‐city children's hospital and was open to children's nurses from the General Medical and Surgical Wards, and the Paediatric Short Stay, Paediatric Liver, and Intensive Care units. Children attending the emergency department following an episode of self‐harm could be admitted to any of the aforementioned wards according to CAMHS bed availability and the nature of the self‐harm.

‘Our Care Through Our Eyes’ was designed by children and young people, registered children's nurses and academics (Manning *et al*. 2015). The digital education RLO consists of three short (15 min) e‐learning packages which consist of a variety of interactive exercises:

1. understanding self‐harm and care pathways for CYP admitted to hospital;
2. effective communication with CYP following self‐harm admission; and
3. assessing risk and managing safety with CYP admitted with self‐harm.

### Participant recruitment

A self‐selected convenience sample was created of registered children's nurses who worked on the specified hospital wards and units and were fluent in the English language. A power calculation, at the outset, indicated 50 nurses would be sufficient to test for a medium sized effect (half a standard deviation difference in pre‐ and postmeasures) at the 5% level (2‐sided test) with power of 80% when the correlation between pre‐ and postmeasures is r = 0.25 or higher. A degree of correlation was factored into the sample size calculation because some participants will have responded on both occasions. The required size was not reached therefore statistical testing was not undertaken.

A Participant Information Sheet (PIS) was distributed to ward managers who promoted the study. Nurses could request further information via email or face‐to‐face. Those nurses who were interested in participating were emailed a link. Participants gave their consent on the preintervention questionnaire.

### Data collection

Quantitative measures in the form of pre‐ and postintervention questionnaires were used to evaluate outcome measures related to nurses' attitudes and behaviours. Whilst Manning *et al*. (2017) assessed attitudes, knowledge, and confidence, this study also assessed empathy, anxiety and beliefs. Four instruments were used for these purposes:

- Attitudes ‐ the Self‐Harm Antipathy Scale (SHAS) (Patterson *et al*. 2007) was used to assess attitudes towards people who self‐harm and has excellent overall internal consistency (Cronbach's alpha of 0.85). The SHAS is a 23‐item self‐report measure, rated on seven‐point Likert scale that consists of six sub‐scales competence appraisal (7 items), care futility (5), client intent manipulation (4), acceptance and understanding (3), rights and responsibilities (2), and needs function (2).
- Beliefs (locus of control) ‐ Rotter's Internal and External (I‐E) locus of control scale (Rotter 1966, Rotter, 1975) was used to assess the extent to which individuals believe their actions can influence outcomes. This scale has very good internal consistency (Cronbach's alpha of 0.72). It consists of 23 forced choice questions (internal v. external statement) yielding a total score ranging from 0 to 23. Higher scores are representative of participants demonstrating inconsistent views about the degree to which they control their own fate (Rotter 1966, 1975). Rotter states people with strong external locus of control tend to praise or blame external factors such as their employer or systems for their fate (Rotter 1966, 1975).
- Empathy ‐ The Reynolds Empathy Scale (RES) (Reynolds 2000) was used to assess levels of nursing empathy as a key component of quality care. This scale has good internal consistency (Cronbach's alpha of 0.64), and consists of 12 items rated on seven‐point rating scale, half of the items are considered to be ‘high empathy’, and the other half to be ‘low empathy’.
- Anxiety and confidence ‐ The Nursing Anxiety and Self‐Confidence with Clinical Decision Making scale (NASC‐CDM) © (White 2014) consists of 27 items rated on six‐point Likert scale that are grouped under three dimensions: using resources to gather information and listening fully, using information to see the big picture, and knowing and acting. Each dimension has a self‐confidence and anxiety component. Excellent internal consistency reported for the three dimensions (Cronbach's alpha 0.86–0.91). The higher the score the higher the self‐confidence or anxiety.

Demographic information was collected via a short questionnaire and three open ended questions were added to the postintervention questionnaire to gain insight into participants' experience of completing the RLO. The questions sought information on the difficulties participants had experienced when caring for CYP who self‐harmed, what had been most beneficial about the RLO intervention and why, and their recommendations to improve the training.

Invitations to the online preintervention questionnaire were circulated to interested participants via email and made available for 4 weeks. At week 5, an invitation to the digital education RLO was circulated via email and this was made available for the following 4 weeks. Participants who agreed to participate in the study completed the prequestionnaire, which took 10–20 min to complete prior to viewing the digital education RLO (Time (T) 1). After completing the e‐learning packages on digital education RLO (T2), participants were asked to complete the same questionnaire (T3) which was made available for 2 weeks following the final deadline for completing the digital education RLO.

The qualitative feedback element of the study (T3) aimed to capture the views and experiences of completing the digital education RLO among participants. Three open ended questions were added to the postintervention questionnaire:

*1*. *Which part of caring for children and young people who have self‐harmed do you find most difficult?*

*2*. *Which part of the RLO intervention did you find most beneficial and why?*

*3*. *What would you recommend to improve the training?*

### Data analysis

Completions rates varied by instrument: SHAS pre *n* = 39, post *n* = 22; LOC pre *n* = 35 post *n* = 22; RES pre *n* = 35 post *n* = 22; NASC‐CDM self‐confidence pre *n* = 33 post *n* = 22, anxiety pre *n* = 33 post *n* = 22. Most instrument items were completed by participants. Instrument scores were calculated by computing the mean of the non‐missing values and multiplying by the number of items.

Demographic variables were analysed and presented using descriptive statistics. The mean and standard deviation of pre‐ and postintervention scores, the mean difference with 95% confidence intervals were calculated for all outcomes. Cronbach's α was calculated for all scales and subscales using the combined pre‐ and posttraining data. All analyses were performed using Statistical Package for the Social Sciences software (SPSS, IBM, Version 26.0).

Answers derived from the open comments were coded and analysed thematically using Ritchie and colleagues thematic analysis framework (Ritchie *et al*. 2003). The framework supported the researcher in generating concepts and themes essential to the research questions/focus and provided a clear structure to the analysis process. A thematic framework was developed and used to classify and organize data from the open comments. This comprised of a series of main themes subdivided by related topics. The thematic framework was then used to index the open comments and then a matrix was created to summarize what was said within each of the themes for each open comment.

### Ethics

Ethical approval was granted for the study by the Health Research Authority on 1 March 2019 (IRAS Project ID: 255760).

## RESULTS

Participant characteristics are presented before considering pre–postintervention scores for the four instruments. Changes to individual items within each instrument are also highlighted.

### Participant characteristics

A total of 42 participants were recruited from a pool of 163 children's nurses across the hospital wards, giving an initial response rate of 26%. The majority of participants were female (*n* = 35, 83%) aged 25–34 years (*n* = 28, 67%) and educated to degree level (*n* = 28, 67%). All participants were either nursing band 5 (*n* = 22, 52%), band 6 (n = 16, 38%), or band 7 (*n* = 4, 10%). Demographics were only recorded pretraining because of the need to maintain anonymity so age, gender and seniority could not be used to adjust the change in means. An exploratory linear regression using age, gender, and seniority (NHS Bands) as independent variables found that being more senior (Band 6/7 vs Band 5) was associated with greater pretraining confidence for Dimension 2 (adjusted mean 4.76 vs 4.13, *P* = 0.029) and Dimension 3 (adjusted mean 4.47 vs 3.92, *P* = 0.033). Age (*P* = 0.048) was associated with SHAS competence appraisal. Those aged 45+ had lower scores than younger age‐groups. Gender was not associated with any of the outcome measures pretraining.

### Outcome measures

Table 1 presents total pre–postintervention scores for each instrument together with mean differences and 95% confidence intervals. Cronbach's α reached an acceptable level (≥0.7) for SHAS overall (0.85), the competence appraisal (0.84), and client intent manipulation (0.80) subscales of SHAS and Rotter's internal and external locus of control scale (0.72). The Cronbach's α for SHAS care futility (α = 0.61), acceptance and understanding (α = 0.50), right and responsibilities (α = 0.67) and needs function (α = 0.44), and the Reynold's Empathy Scale (α = 0.64) were <0.70. The three dimensions (confidence, anxiety) of the NASC‐CSM produced Cronbach's α's in the range 0.86 to 0.91.

**TABLE 1:** Pre–postintervention outcome scores

Attribute	Instrument (items)	Cronbach's α	Prescore Mean (SD)	Postscore Mean (SD)	Pre–postscore difference Mean (95% CI)
Attitudes	Self‐harm antipathy scale (SHAS)		(*n* = 39)	(*n* = 22)	
	Overall total (30)	0.85	68.1 (13.3)	62.8 (17.3)	5.5 (−2.4 to 13.4)
	*Subscales*:				
	Competence appraisal (7)	0.84	15.2 (5.0)	13.7 (6.0)	1.4 (−1.5 to 4.3)
	Care futility (5)	0.61	8.2 (2.7)	8.3 (3.0)	−0.1 (−1.6 to 1.4)
	Client intent manipulation (4)	0.80	9.0 (3.9)	7.7 (3.7)	1.3 (−0.7 to 3.4)
	Acceptance and understanding (3)	0.50	5.5 (1.8)	5.1 (1.9)	0.4 (−0.6 to 1.4)
	Rights and Responsibilities (2)	0.67	9.4 (2.9)	8.3 (3.3)	1.1 (−0.6 to 2.7)
	Needs function (2)	0.44	4.7 (1.4)	3.9 (1.9)	0.8 (−0.1 to 1.6)
			(*n* = 35)	(*n* = 22)	
Beliefs (locus of control)	Rotter's internal and external locus of control scale (LOC) (23)	0.72	12.3 (5.6)	13.4 (3.3)	−1.1 (−3.4 to 1.2)†
Empathy	Reynolds empathy scale (RES) (12)	0.64	56.0 (5.5)	57.1 (5.6)	−1.1 (−4.1 to 1.9)
Anxiety and confidence	Nursing anxiety and self‐confidence with clinical decision‐making scale (NASC‐CDM) ©		(*n* = 33)	(*n* = 22)	
	*Dimension 1*				
	Confidence (13)	0.90	4.4 (0.6)	4.7 (0.7)	−0.2 (−0.6 to 0.1)
	Anxiety (13)	0.91	1.9 (0.5)	2.0 (0.7)	−0.1 (−0.5 to 0.2)
	*Dimension 2*				
	Confidence (7)	0.91	4.0 (0.8)	4.2 (0.8)	−0.2 (−0.6 to 0.3)
	Anxiety (7)	0.90	2.3 (0.6)	2.6 (0.9)	−0.3 (−0.7 to 0.1)
	*Dimension 3*				
	Confidence (7)	0.86	3.9 (0.7)	4.1 (0.8)	−0.2 (−0.6 to 0.2)
	Anxiety (7)	0.86	2.7 (0.6)	2.6 (0.8)	0.1 (−0.3 to 0.5)

^†^ Confidence interval was calculated using unequal variances.

SHAS overall mean total reduced following training from 68.1 to 62.8, and a reduction was observed for most of the SHAS sub‐scales although these were small. There were small changes in mean scores for LOC from 12.3 to 13.4 and for RES from 56.0 to 57.1. NASC‐CDM mean scores were similar before and after training.

#### Attitudes

Five items in the SHAS (Patterson *et al*. 2007) reflect positive attitudes towards patients who self‐harm. For example, item 25 invites respondents to rate their agreement with the statement *‘I help self‐harming patients feel positive about themselves’*. Pretest, 20/39 (51%) of participants ‘strongly agreed’ or ‘agreed’ with this statement increasing to 16/22 (73%) posttest. Small increases in participants' positive attitudes towards patients who self‐harm were evident for the other four items.

Nine items in the SHAS reflect negative attitudes towards people who self‐harm. For example, item 4 invites respondents to rate the statement *‘Self‐harming clients do not respond to care’*. Pretest, 33/39 (85%) of participants ‘disagreed’ or ‘strongly disagreed’ with this statement, increasing to 20/22 (91%) posttest. Item 1 states that *‘People who self‐harm are usually trying to get sympathy from others’*. Pretest, 7/39 (18%) ‘Strongly disagreed’ with this statement, which increased to 10/22 (46%) posttest. Small changes were identified for the other six items.

Two items refer to the function that self‐harm has for an individual. For example, item 12 states that *‘Self‐harm may be a form of reassurance for the individual that they are really alive and human’*. Pretest, 3/39 (8%) of participants ‘Strongly agreed’ with the statement, which increased to 9/22 (41%) posttest. Collectively, these findings demonstrate that following the training there were small increases in participants' empathy and positive attitudes, small decreases in their negative attitudes, and an improved understanding of the function that self‐harm fulfils for individuals.

#### Beliefs (locus of control),

The pre‐ and posttraining mean LOC scores (12.3 and 13.4, respectively) were mid‐way on the 0–23 total score range and suggests nurses demonstrate inconsistent views about the degree to which they control their own actions.

#### Empathy

There were mixed responses to individual items in the RES (Reynolds 2000). Five items reflect positive empathic behaviours including active listening and being sensitive to the feelings of others. For example, item 7 invites respondents to rate the degree to which they are like the statement *‘Responds to feelings and meanings’*. Pretest, 19/35 (54%) of participants rated themselves as being ‘nearly always like’ or ‘always like’ the statement, which increased to 17/22 (77%) posttest. Small improvements in positive empathic behaviours were evident for the other four items.

Six items in the RES reflect negative empathic behaviours such as manipulative and hostile communications. For example, item 8 invites respondents to rate the degree to which they are like the statement *‘Interrupting and seeming in a hurry’*. Pretest, 9/35 (26%) of participants rated themselves as being ‘never like’ the statement, which decreased to 3/22 (14%) posttest. Small negative changes to participants' ratings of their empathic behaviour were evident for four of the five of the other items.

#### Anxiety and self‐confidence

The majority of NASC‐CDM © scale dimension 1 items revealed small improvements in self‐confidence posttest. However, lower self‐confidence scores were evident across three items: recognizing the need to review a protocol or procedure, correlating physical assessment findings with a client's non‐verbal cues, and recognizing the need to talk to an instructor or staff about interventions. Anxiety scores for dimension 1 were higher postintervention demonstrating small increases in anxiety. Three items revealed no discernible pre–post change: ability to use active listening skills, ability to realize the need for guidance on intervention options, and ability to remain open to different reasons for a patient's problem.

Dimension 2 item all showed small improvements in self‐confidence posttest except for one: ability to recall relevant knowledge learned in the past. Anxiety scores improved in only two items, remained similar in one and worsened in four including ability to easily see important patterns in information, and ability to see the full clinical picture of the patient's problem. Dimension 3 items all showed small improvements in self‐confidence posttest. Anxiety scores showed small reductions in anxiety for two items however overall, the results demonstrate a small increase in nursing anxiety postintervention.

### Qualitative feedback

Nineteen of the 22 participants who completed the training also provided qualitative feedback. They responded to three questions concerning barriers to caring for CYP who self‐harm, impact of the training, and recommendations for future training.

#### Barriers to care

All but four of the participants stated that a lack of confidence in themselves and in their knowledge and understanding of self‐harm were barriers to care. A particular challenge was initiating conversations because participants were unsure whether CYP wanted to discuss the reasons behind their act of self‐harm. However, seven participants also acknowledged that they struggled to understand and listen to the reasons why CYP self‐harmed. More broadly, participants were concerned that the care they could offer would not be adequate or effective.

*‘I would be worried that interventions … to distract or just talk to them would not be good enough to help them’* (Sister)

Two respondents also raised time pressures within their work context that restricted opportunities to ‘*build rapport and trust’* with CYP. One respondent reported a lack of support from mental health professionals.

#### Impact of the training

All participants found elements of the training beneficial to their practice, particularly the resources that could be accessed online. Tools that demonstrated techniques in initiating conversations and listening were highly rated by many participants.

*‘I liked that you were able to print off the different techniques which you could use to approach conversations with CYP and their families*. *The videos were also useful to see real conversations which CYP found to be helpful or not helpful’* (Staff Nurse)

#### Recommendations

Few recommendations were made to improve the training although one participant suggested that face‐to‐face and scenario‐based training may be beneficial. Other comments pertained to the questionnaires. Some participants had found individual items irrelevant and confusing.

## DISCUSSION

Despite the increasing rates of CYP self‐harm with subsequent emergency department attendances (Nuffield Trust 2020) and hospital admissions (NSPCC 2016) there are limited training initiatives available to support children's nurses caring for these patients. Only one training programme was found that was specific to the individual needs of children's nurses caring for patients who are admitted following an episode of self‐harm in acute paediatric inpatient settings (Manning *et al*. 2017). Until now, the RLO training programme created by Manning *et al*. (2017) had only been evaluated in a single centre site in Nottingham. This current study implemented and evaluated RLO training programme with children's nurses working in a different setting with a new set of outcome measures in order to further evaluate the training programmes effectiveness and efficiency.

Antipathy decreased overall from pre‐ to posttraining and this was observed across nearly all the SHAS sub‐scales. There were small increases in locus of control, empathy and self‐confidence, and a small increase in anxiety. Locus of control was mid‐way on the 0–23 range and suggests nurses demonstrate inconsistent views about the degree to which they control their own actions. They probably believe they do control their own fate in some areas of working life, while believing that they have little control in other areas. There was a mixed picture in terms of improvement at the individual item level of the four scales.

Manning *et al*. (2017) found similar results when observing the outcome measures attitudes and confidence. Lack of confidence and anxiety is a common theme in the literature (Bruder *et al*. 2017; Carter *et al*. 2018; Leddie *et al*. 2021; Pryjmachuk *et al*. 2012; Ravenna & Cleaver 2016) which needs to be managed. It is important that children's nurses are supported in their knowledge and possess the skills concerning mental health problems because they are at forefront of care for CYP who self‐harm.

There were both small increases in effective behaviours and small increases in ineffective behaviours relating to empathy. Improvements in nurse's empathy were found following the RLO intervention based on open‐ended comments: nurses reported a better understanding of, and reasons why, CYP self‐harm. No improvements in nurse's beliefs/controls were found and the results for nurse's anxiety were mixed and thus inconclusive. No other study has yet measured empathy in this particular setting and as such no comparison can be made. However, a small number of studies (Carter *et al*. 2018; Chapman & Martin 2014; Dickinson & Hurley 2012; Leddie *et al*. 2021) highlighted how children nurses may benefit from some empathy and attitudes‐based training to assimilate concepts, promote communication, and may increase the delivery of compassionate and effective care to CYP self‐harm. These studies (Pryjmachuk *et al*. 2012; Thomas 2017; Wissow *et al*. 2011) illustrated the importance of empathy training for children's nurses when dealing with CYP who self‐harm or a suicide attempt and found that nurses who complete attitudes training adopt a more patient‐centred and empathetic response to psychosocial distress.

Findings from the qualitative analysis supported the findings from the literature review (Carter *et al*. 2018; Leddie *et al*. 2021; Little, 2015; Thomas 2017 Vallières‐Noël *et al*. 2016). These studies illustrated how children's nurses caring for CYP who self‐harm lack confidence in themselves, their knowledge and understanding of self‐harm and often fear they are *‘not good enough’*. The children's nurses in this study reported the RLO intervention was beneficial to their practice and reported improved knowledge and understanding of self‐harm. They valued the re‐usable communication tools and reflective scenario videos as key components of the training. These findings support Manning *et al*. (2017) methodological development of the RLO intervention. E‐learning that is co‐designed with stake‐holders and is tailored to the needs the nursing profession is more likely to prove successful in increasing knowledge as well as being accepted and reused.

The COVID‐19 pandemic has changed how education is delivered and this includes how nurses participate in the continued professional development programmes to update their skills and education in health care settings. We argue that children's nurses' access to continuing professional development could be made more attainable, realistic and relevant. The use of e‐learning has surged since COVID‐19. The distinctive rise of e‐learning whereby teaching is undertaken remotely and on digital platforms has emerged as a popular tool to provide flexible, learner‐centered training to keep nurses' knowledge, and skills up‐to‐date (Green & Huntington 2017; Meredith *et al*. 2018; Mlambo *et al*. 2021). E‐learning allows nurses to learn at their own pace, permits access for rotating rosters and people living in geographically‐diverse regions in a cost‐effective and timely manner (Jorm *et al*. 2010; Lee *et al*. 2013; Meredith *et al*. 2018). Studies (Jorm *et al*. 2010; Mlambo *et al*. 2021; Rouleau *et al*. 2017) have reported the efficacy of e‐learning packages in mental health training as comparable to face to face training. In addition, e‐learning is recognized as a cost‐effective method that produces positive outcomes. Within the current political climate, continued economical efforts must be made to support children's nurses with their on‐going development (Meredith *et al*. 2018). This is imperative to ensure children's nurses are equipped with appropriate knowledge and skills to deliver a high standard care to CYP with mental health problems.

### Strengths and limitations

This study shows how it would be possible to measure the impact of a RLO intervention delivered via e‐learning using a selection of prevalidated tools that measure empathy, beliefs (locus of control), self‐confidence, and anxiety. Including a diverse sample of children nurses from a broad range of pay bandings, age and level of education, ensured that children's nurses views of the RLO intervention were adequately represented. The nurse characteristics were also similar to those in the original study undertaken by Manning *et al*. (2017) and helped in terms of comparability.

Although this study was able to provide new important data several limitations are noted. Approximately 25% of children's nurses were recruited to the study and they may not be representative of the site population. The final sample size was underpowered for the purposes of the statistical testing and so summary statistics including mean difference with 95% confidence interval were reported. Pretraining completion rates varied by instrument from 39 who completed SHAS to 33 who completed NASC‐CDM. The posttraining questionnaire was completed by 22 participants. Some of the changes observed could therefore be down to response bias. The study was a master's dissertation and there were time constraints for the data collection period which may have affected participation rates. In addition, another mandatory research study was running simultaneously on one of the wards which may have impacted nurses' desires to complete the training. Also, it was not possible to link pre‐ and postintervention responses to the same nurse because of the need to maintain anonymity. The authors suggest that future researchers seek methodological advice on the possibility of setting up a data collection tool that can link a person's data while ensuring the researchers remain blinded. The Reynold's Empathy Scale and four of the SHAS sub‐scales (care futility, acceptance and understanding, rights and responsibilities, needs function) did not meet the reliability threshold (Cronbach's α ≥ 0.7) and suggests that further testing and validation is required. Some of the SHAS subscales consisted of only two to three items (acceptance and understanding (3), rights and responsibilities (2), and needs function (2)) which may limit their usefulness.

The lack of a control group also limited the ability to make any claims regarding causation. In addition, recruitment took place from a single site and thus it is difficult to make generalizations to other registered children's nurses working with CYP who self‐harm. However, the findings could be used to inform further investigations. Future research should be conducted over a more extended timescale to demonstrate the effectiveness of the RLO intervention over time.

Further improvements may be required to make the ‘Our Care Through Our Eyes’ intervention more effective. Introducing face‐to‐face sessions and scenario‐based training were improvements suggested by the participants. In addition, participants reported some of the questionnaires to be ‘confusing and ambiguous’. A future study may benefit from designing a set of specific questionnaires for the study. However due to time restrictions prevalidated questionnaires were used in this study.

## CONCLUSION

Evaluation of the RLP (Manning *et al*. 2017) self‐harm e‐learning has shown the possibility of improving children's nurses attitudes, beliefs (locus of control), empathy, and confidence when caring for CYP who self‐harm. As previously highlighted CYP admitted to hospital for self‐harm or attempted suicide can present significant difficulties on paediatric wards in terms of care management and safety provision. Children's nurses have raised concerns about the suitability of admission to paediatric wards. The reasoning behind these concerns relate to a lack of expertise, knowledge, confidence and unmet training needs. Confidence and education are closely related, and it is likely that education and training opportunities will improve children's nurses understanding and confidence in assessing and managing self‐harm which is fundamental for CYP with self‐harm. Overall, mental health training (including self‐harm) must continue to be implemented in both preregistration and postregistration education to ensure children's nurses are well equipped to care for the children and young people of future generations.

### RELEVANCE TO PRACTICE

The RLO intervention ‘Our Care Through Our Eyes’ described here may be fundamental to the delivery of compassionate and effective care to CYP following self‐harm. This study has demonstrated the potential benefits of this e‐learning programme on improving children's nurses' attitudes, beliefs (locus of control), empathy and confidence when providing care to CYP who self‐harm. Further study should be undertaken to evaluate, via an adequately powered study, the potential to include this intervention in training for children's nurses. Based on the results of this study, it is also recommended that mental health content and clinical learning experiences should be included in undergraduate children's nursing curricula enabling students to provide first assistance to a person experiencing a mental health problem.

## ETHICAL STATEMENT

Ethical approval was obtained from the Health Research Authority on 1st March 2019 (IRAS Project ID: 255760).

## PATIENT CONSENT FOR PUBLICATION STATEMENT

Not applicable

## Data Availability

The data that support the findings of this study are available from the corresponding author upon reasonable request.

## References

[inm13041-bib-0001] Bolland, R. , Richardson, J. & Calnan, R. (2017). How professionals should communicate with children who have mental healthcare needs. Nursing Children and Young People, 29 (1), 20–24. 10.7748/ncyp.2017.e814. 28162062

[inm13041-bib-0002] Bruder, N. , Gettings, S. & Grealish, A. (2017). Engagement and effective information provision to promote recovery from depression. Nursing Children and Young People, 29 (2), 38–43. 10.7748/ncyp.2017.e733. 28262068

[inm13041-bib-0003] Carter, T. , Latif, A. , Callaghan, P. & Manning, J. C. (2018). An exploration of predictors of children's nurses' attitudes, knowledge, confidence and clinical behavioural intentions towards children and young people who self‐harm. Journal of Clinical Nursing, 27 (13–14), 2836–2846. 10.1111/jocn.14361. 29569381

[inm13041-bib-0004] Chapman, R. & Martin, C. (2014). Perceptions of Australian emergency staff towards patients presenting with deliberate self‐poisoning: A qualitative perspective. International Emergency Nursing, 22 (3), 140–145. 10.1016/j.ienj.2014.03.002. 24768529

[inm13041-bib-0005] Dickinson, T. & Hurley, M. (2012). Exploring the antipathy of nursing staff who work within secure healthcare facilities across the United Kingdom to young people who self‐harm. Journal of Advanced Nursing, 68 (1), 147–158. 10.1111/j.1365-2648.2011.05745.x. 21711386

[inm13041-bib-0006] Frith, E. (2017). Access and Waiting Times in Children and Young people's Mental Health Services. London: Education Policy Institute.

[inm13041-bib-0007] Geulayov, G. , Casey, D. , McDonald, K. C. *et al*. (2018). Incidence of suicide, hospital‐presenting non‐fatal self‐harm, and community‐occurring non‐fatal self‐harm in adolescents in England (the iceberg model of self‐harm): A retrospective study. The lancet. Psychiatry, 5 (2), 167–174. 10.1016/S2215-0366(17)30478-9. 29246453

[inm13041-bib-0008] Green, J. K. & Huntington, A. D. (2017). Online professional development for digitally differentiated nurses: An action research perspective. Nurse Education in Practice, 22, 55–62. 10.1016/j.nepr.2016.11.009. 27940391

[inm13041-bib-0009] Hawton, K. , Saunders, K. E. & O'Connor, R. C. (2012). Self‐harm and suicide in adolescents. Lancet (London, England), 379 (9834), 2373–2382. 10.1016/S0140-6736(12)60322-5. 22726518

[inm13041-bib-0010] Higson, J. , Emery, A. & Jenkins, M. (2017). Improving children's nurses' knowledge of caring for people with mental health problems. Nursing Children and Young People, 29 (1), 25–29. 10.1111/jocn.14361. 28162082

[inm13041-bib-0011] Jorm, A. F. , Kitchener, B. A. , Fischer, J. A. & Cvetkovski, S. (2010). Mental health first aid training by e‐learning: A randomized controlled trial. The Australian and New Zealand Journal of Psychiatry, 44 (12), 1072–1081. 10.3109/00048674.2010.516426. 21070103

[inm13041-bib-0012] Karman, P. , Kool, N. , Poslawsky, I. E. & van Meijel, B. (2015). Nurses' attitudes towards self‐harm: A literature review. Journal of Psychiatric and Mental Health Nursing, 22 (1), 65–75. 10.1111/jpm.12171. 25490929

[inm13041-bib-0013] Kessler, R. C. , Berglund, P. , Demler, O. , Jin, R. , Merikangas, K. R. & Walters, E. E. (2005). Lifetime prevalence and age‐of‐onset distributions of DSM‐IV disorders in the National Comorbidity Survey Replication. Archives of General Psychiatry, 62 (6), 593–602. 10.1001/archpsyc.62.6.593. 15939837

[inm13041-bib-0014] Leddie, G. , Fox, C. & Simmonds, S. (2021). Nurses' experiences of working in the community with adolescents who self‐harm: A qualitative exploration. Journal of Psychiatric and Mental Health Nursing, 00, 1–11. 10.1111/jpm.12806. 34797016

[inm13041-bib-0015] Lee, Y. H. , Hsieh, Y. C. & Chen, Y. H. (2013). An investigation of employees' use of E‐learning systems: Applying the technology acceptance model. Behaviour & Information Technology, 32 (2), 173–189. 10.1080/0144929X.2011.577190.

[inm13041-bib-0016] Little, M. C. (2015). An exploration of children's nursing graduates' ability to assess children's emotional health and well-being. Journal of child health care: for professionals working with children in the hospital and community, 19 (3), 370–380. 10.1177/1367493513509032. 24270990

[inm13041-bib-0017] Lowe, L. & Campbell, A. (2014). Evaluation of a study day on child and adolescent mental health services. Mental Health Practice, 17 (5), 19–24. 10.7748/mhp2014.02.17.5.19.e826.

[inm13041-bib-0018] Manning J.C , Carter T. , Latif A. (2015). ‘Our Care Through Our Eyes’. Available at: http://sonet.nottingham.ac.uk/rlos/mentalhealth/octoe/knowledge/resources.html (accessed 11 May 2021)

[inm13041-bib-0019] Manning, J. C. , Carter, T. , Latif, A. *et al*. (2017). Our care through our Eyes'. Impact of a co‐produced digital educational programme on nurses' knowledge, confidence and attitudes in providing care for children and young people who have self‐harmed: A mixed‐methods study in the UK. BMJ Open, 7 (4), e014750. 10.1136/bmjopen-2016-014750. PMC5623397 28473515

[inm13041-bib-0020] Mental Health Foundation (2015). Fundamental Facts about Mental Health. London: Mental Health Foundation.

[inm13041-bib-0021] Meredith, P. , Yeates, H. , Greaves, A. *et al*. (2018). Preparing mental health professionals for new directions in mental health practice: Evaluating the sensory approaches e‐learning training package. International Journal of Mental Health Nursing, 27 (1), 106–115. 10.1111/inm.12299. 28042908

[inm13041-bib-0022] Mlambo, M. , Silén, C. & McGrath, C. (2021). Lifelong learning and nurses' continuing professional development, a metasynthesis of the literature. BMC Nursing, 20 (1), 62. 10.1186/s12912-021-00579-2. 33853599 PMC8045269

[inm13041-bib-0023] Morgan, C. , Webb, R. T. , Carr, M. J. *et al*. (2017). Incidence, clinical management, and mortality risk following self harm among children and adolescents: Cohort study in primary care. BMJ, 359, j4351. 10.1136/bmj.j4351. 29046278 PMC5641980

[inm13041-bib-0024] National Institute for Health and Care Excellence (2004). Self‐harm in over 8s: short‐term management and prevention of recurrence: cg16 . Available at: www.nice.org.uk/guidance/cg16 (accessed 11 May 2021) 31891461

[inm13041-bib-0025] NHS Digital (2020). Mental health of children and young people in England, 2020: Wave 1 follow up to the 2017 survey. Available at: Mental Health of Children and Young People in England, 2020: Wave 1 follow up to the 2017 survey‐NHS Digital

[inm13041-bib-0026] NSPCC (2016). Rise in children hospitalised for self‐harm as thousands contact Childline. Last accessed March 2018: https://www.bbc.com/news/health‐38252335

[inm13041-bib-0027] Nuffield Trust (2020). Hospital admissions as a result of self‐harm in children and young people. Available at: Hospital admissions as a result of self‐harm in children and young people. The Nuffield Trust (accessed 11 May 2021).

[inm13041-bib-0028] Patterson, P. , Whittington, R. & Bogg, J. (2007). Measuring nurse attitudes towards deliberate self‐harm: The self‐harm antipathy scale (SHAS). Journal of Psychiatric and Mental Health Nursing, 14 (5), 438–445. 10.1111/j.1365-2850.2007.01102.x. 17635251

[inm13041-bib-0029] Pryjmachuk, S. , Graham, T. , Haddad, M. & Tylee, A. (2012). School nurses' perspectives on managing mental health problems in children and young people. Journal of Clinical Nursing, 21 (5–6), 850–859. 10.1111/j.1365-2702.2011.03838.x. 21883575

[inm13041-bib-0030] Public Health England . (2017). Intentional self‐harm in adolescence: An analysis of data from the Health Behaviour in School‐aged Children (HBSC) survey for England, 2014. Available at: Main heading (publishing.service.gov.uk) (accessed 11 may 2021)

[inm13041-bib-0031] Ravenna, J. & Cleaver, K. (2016). School Nurses' experiences of managing young people with mental health problems: A scoping review. The Journal of school nursing: the official publication of the National Association of School Nurses, 32 (1), 58–70. 10.1177/1059840515620281. 26656230

[inm13041-bib-0032] Reynolds, J. W. (2000). The Measurement and Development of Empathy in Nursing. Aldershot: Ashgate.

[inm13041-bib-0033] Richardson, B. P. (2011). Child and adolescent mental health service (CAMHS) placements for pre‐registration child branch nursing students: Potential benefits. Nurse Education Today, 31 (5), 494–498. 10.1016/j.nedt.2010.08.007. 20837375

[inm13041-bib-0034] Ritchie, J. , Spencer, L. & O'Connor, W. (2003). Carrying out qualitative analysis. In: J. Ritchie & L. Spencer (Eds). Q*ualitative research practice: A guide for social science students and researchers* , Vol. 2003. (pp. 219–262). London: Sage.

[inm13041-bib-0035] Rotter, J. B. (1966). Generalized expectancies for internal versus external control of reinforcement. Psychological Monographs: General and Applied, 80 (1), 1–28. 10.1037/h0092976. 5340840

[inm13041-bib-0036] Rotter, J. B. (1975). Some problems and misconceptions related to the construct of internal versus external control of reinforecement. Journal of consulting and clinical psychology, 43, 56–67. 10.1037/h0076301.

[inm13041-bib-0037] Rouleau, G. , Gagnon, M. P. , Côté, J. , Payne‐Gagnon, J. , Hudson, E. & Dubois, C. A. (2017). Impact of information and communication technologies on nursing care: Results of an overview of systematic reviews. Journal of Medical Internet Research, 19 (4), e122. 10.2196/jmir.6686. 28442454 PMC5424122

[inm13041-bib-0038] Rowe, S. L. , French, R. S. , Henderson, C. , Ougrin, D. , Slade, M. & Moran, P. (2014). Help‐seeking behaviour and adolescent self‐harm: A systematic review. The Australian and New Zealand Journal of Psychiatry, 48 (12), 1083–1095. 10.1177/0004867414555718. 25335872

[inm13041-bib-0039] Thomas, L. (2017). Nursing children and young people: What mental health training is required? British Journal of Nursing, 26 (4), 234–237. 10.12968/bjon.2017.26.4.234. 28230432

[inm13041-bib-0040] UK Parliament . (2018). Mental illness: Children and young people. Question for Department of Health and Social Care. Retrieved from: Written questions and answers ‐ Written questions, answers and statements – UK Parliament

[inm13041-bib-0041] Vallières‐Noël, M. M. , Garçon, S. , Rosmus, C. , Goulnik, F. & Lavoie‐Tremblay, M. (2016). Exploring the needs for support of pediatric nurses caring for children with a mental health disorder hospitalized in non‐psychiatric units. Archives of Psychiatric Nursing, 30 (2), 170–177. 10.1016/j.apnu.2015.08.008. 26992867

[inm13041-bib-0042] von Elm, E. , Altman, D. G. , Egger, M. *et al*. (2007). Strengthening the reporting of observational studies in epidemiology (STROBE) statement: Guidelines for reporting observational studies. BMJ, 335 (7624), 806–808. 10.1136/bmj.39335.541782.AD. 17947786 PMC2034723

[inm13041-bib-0043] White, K. A. (2014). Development and validation of a tool to measure self‐confidence and anxiety in nursing students during clinical decision making. The Journal of Nursing Education, 53 (1), 14–22. 10.3928/01484834-20131118-05. 24256004

[inm13041-bib-0044] Wissow, L. , Gadomski, A. , Roter, D. , Larson, S. , Lewis, B. & Brown, J. (2011). Aspects of mental health communication skills training that predict parent and child outcomes in pediatric primary care. Patient Education and Counseling, 82 (2), 226–232. 10.1016/j.pec.2010.03.019. 20444568 PMC2947561

